# If People Are Attached to Plants, Do They Love Other People? Case of the Russian Youth

**DOI:** 10.3390/bs10020040

**Published:** 2020-01-22

**Authors:** Sofya Nartova-Bochaver, Elena Muhortova

**Affiliations:** 1 Department of Psychology, National Research University Higher School of Economics, 101000 Moscow, Russia; 2 Department Psychology of Education, Moscow State University of Psychology and Education, 121500 Moscow, Russia; muhortova.elena@yandex.ru

**Keywords:** plant world, moral, motive, students

## Abstract

People’s attachment to the plant world makes a great contribution to the maintenance of psychological well-being. At the same time, little is known regarding the contribution of attitudes to plants to people’s morality; the current study is aimed at filling this gap. We assumed that the more positive the attitude to plants is, the higher the level of moral motives is. The survey was conducted on the Russian sample; 257 participants (students from Moscow universities, 199 female, M_age_ = 21.1, SD_age_ = 2.5) were recruited. The following tools were used: a questionnaire People and Plants (PaP) consisting of five sub-scales (joy, esthetics, practice, closeness to nature, and ecology) and Moral Motives Model scale (MMM scale) including six sub-scales (self-restraint, not harming, social order, self-reliance (industriousness), helping/fairness, and social justice). It was found that all parameters of the positive attitudes to plants, except practice, were strongly positively connected with moral motives. Multi-regression analysis allowed developing certain models demonstrating the contribution of attachment to the plant world to people’s morality. The proscriptive motives (especially self-restraint) are more sensitive to attitudes to flora as compared to prescriptive motives; prescriptive motive self-reliance was not predicted by the attitude to flora at all. Moreover, the findings seem to be gender-sensitive (predictions are higher in females). The obtained results are discussed referring to the reverence for life ethics by Schweitzer, deep ecology by Næss, biophilia hypothesis by Wilson, and psychology of moral expansiveness by Crimston et al.

## 1. Introduction

The relationship between a person and nature has been attracting more and more of researchers’ attention during the last decades for many reasons: search for natural self-help techniques, environmental goals, etc. The current research is dedicated to investigating whether people with positive attitudes to the plant world are more moral compared with those who are not attached to plants. The contemporary psychology usually divides and even opposes the social and natural in personal lives and communication. In this paper, we wanted to see whether (and how) such very distant subjects as the relation to plants (natural variable) and moral motives (social variable) relate to each other.

The current research is based on the following theoretical sources. First of all, there is the reverence for life theory by Schweitzer [1], maintaining that the only thing we are sure of is that we live and want to go on living. Moreover, we share this desire with everything else that lives, and we have to respect it as we would like to be recognized. According to Schweitzer, the ethical will-to-live is an innate element of each person that commands him/her to respect all wills-to-live as his/her own, equally. Ethics was defined as responsibility without limit towards all that lives. In the long run, every human action should be directed toward the preservation and promotion of one’s own life and the life of the creatures that are part of one’s environment. 

Being a realist, Schweitzer understood that people are forced to exploit or even kill other living creatures to survive; to save one life, they are often obliged to destroy another one. To avoid the intrapersonal conflict, Schweitzer created the following guideline: whenever people injure life of any sort, they must be quite clear whether it is necessary. They must never do it needlessly, not even with what seems insignificant. 

Nevertheless, the most harshly criticized part of Schweitzer’s philosophy was his refusal to rank the life forms. In this regard, Schweitzer was blamed for pantheism, anthropomorphism, and guilt mongering. Schweitzer advocated that all life is sacred. His support for the intrinsic value of all living beings originates in his metaphysics, which suggests there is a cosmic, creative, and mysterious will-to-live, in which all Being is grounded. He did not permit ranking of life forms, even though he did not de facto attribute the same value to all living creatures. There arises the question of whether it is the task of ethics to distinguish between different life forms and to decide which ones deserve more or less protection. 

At the same time, based on this idea, Schweitzer suggested another moral “skill” that would help people both survive and maintain their self-respect—that is, recognition of the equality of different living forms’ rights. Thanks to this belief, people, on the one hand, tend to appreciate other species’ contribution to their survival, and, on the other hand, are condemned to feel a pang of so-called existential guilt which, in turn, awakens moral elevation in them. The most valuable consequence from Schweitzer’s theory is that, striving for civilization, people must respect the nature holistically, and this may help them appreciate other people and be respected by them, in turn. Thus, environmental attitudes towards life and ethical stances towards society are interrelated.

Then, there is deep ecology by Næss [2], arguing that through the process of self-actualization, one transcends the notions of “egoistic” Self and arrives at the position of an ecological self. Deep ecology’s main principle is the belief that the living environment, as a whole, should be regarded as having certain fundamental moral and legal rights to live and flourish, independent of its practical benefits for human use. This approach is often framed in terms of the idea of a much broader identity and sociality; it recognizes diverse communities of life on Earth that are composed through ethical relations, that is, the valuing of other beings as more than resources for survival only. Usually, this philosophy provides a foundation for the environmental, ecological, and green movements and has fostered a new system of ecological ethics advocating wilderness preservation. At the same time, it emphasizes the fact that life is one but has different forms. That is why the moral boundaries between different species become more transparent, and environmentally responsible people may be altruists as well, being ready to “do good for the other”.

Further, there is the biophilia hypothesis by Wilson [3], stating that the predisposition to form an emotional connection to the natural environment is inherited because such a relationship would have been adaptive in the context within which humans were evolving their defining characteristics. Biophilia, in a broad meaning, is love for life or living systems, an orientation of being attracted to all that is alive and vital. Even though the biophilia hypothesis is usually an essential thing for solving environmental problems, it also contributes to the understanding of humans’ survival and adaptation, and has an active moral component. It suggests that the affiliation to life and its connected processes has conferred significant advantages in human evolution, to the extent that humans have adapted, persisted, and emerged. 

According to Wilson’s co-author and follower Kellert [4], the biophilic tendency takes nine forms of expression: utilitarian, naturalistic, ecological/scientific, aesthetic, symbolic, humanistic, dominant, moralistic and negative. Each form of expression has an evolutionary ground that first favors survival and then the full realization of the Self. Kellert thought that these values might constitute a kind of “genetic inclination” to affiliate with natural processes. A strong sense of affiliation with nature sustains people’s ethical responsibility for the natural world, including both human and non-human species. It is noteworthy that nature may impact on morality directly and indirectly, through the aesthetic attractiveness of nature that, in turn, contributes to morality. Thus, a famous Italian philosopher Capitini noted: “To live near to trees with openness is to receive much more from them than might seem; but this attention must be open and friendly, respectful of these life forms and their exertion; and thus they reciprocate and bring peace” [5] (p. 10).

Now, urbanization has distanced large groups of people from nature, and the ethical bonds people held with the land before have been broken. It may result in losing not only social and psychological benefits received by people from nature previously, but also in losing all profits for their moral and spiritual development [6]. This bond must be recovered for people’s moral and mental health.

Further, according to a new theory of moral expansiveness by Crimston et al. [7], the extent to which people respect others and care for them depends on the distinctions individuals make between entities deemed worthy or unworthy of moral consideration, or people’s ethical boundaries. Thus, people can include or exclude other living beings from their moral responsibility, leading either to expressing empathy with these entities or neglecting them. In the recent paper prepared by Moreton et al. [8], in the paradigm of the moral expansiveness theory, it was shown that moral elevation and nature connectedness, both being kinds of the person’s transcendence, are intimately connected. People who demonstrated higher levels of such emotions, like awe and respect, intended to more likely practice pro-environmental behaviors. However, unlike our study, nature connectedness was considered a dependent variable, and moral elevation an independent one. 

In prehistoric times, interaction with nature was crucial for people’s basic survival. As our species evolved, and the societies became increasingly more complex, they diversified; social, and now even virtual interactions became more critical and sophisticated than human-nature relations, which resulted in the individuals’ feeling of self-alienation. That is why it is of very high importance to awaken in people their sense of belonging to nature. In our opinion, the plant world is the most important and beautiful part of nature. Plants have a special place in life—unlike inanimate objects (for instance, stones and rocks)—plants give people feedback on their care and interest; in contrast to the animal world, plants are not so demanding and intrusive.

Papers are showing a positive contribution of various interactions with plants (walking in parks, gardening, taking care of office plants, etc.) to physical and mental health [9,10]. At the same time, little is known about the “ennobling”, “elevating” function of these interactions, e.g., about whether attachment to plants is positively connected to moral attitudes to other people or not. Previous research has shown, however, that environmental identity is more strongly connected to egoistic than to biospheric and altruistic values, and to natural than social empathy; however, all of these connections are positive [10]. In the brilliant review by Elings [9], it was shown that gardening gave the participants peacefulness, quiet and fascination, helped them maintain positive relations with others, and increased their feeling of purpose in life and self-acceptance. Interaction with in-door plants helped people who experienced burn-out syndrome to rehabilitate and relieve blood pressure, if needed. Some studies show that horticultural therapy can lead to social inclusion. To sum up, the previous results suggest that interaction with plants is related to the presence of such personal features and states that may contribute to changing people’s attitudes to others and a world view, in general, to a less demanding, more tolerant and compassionate one. Based on these results, we assumed that positive attitudes to the plant world may be positively connected with or even contribute to people’s moral motives. 

## 2. Materials and Methods

In this study, 257 students from Moscow universities participated (199 female, M_age_ = 21.1, SD_age_ = 2.1). A convenience sampling was used; participation was anonymous and voluntary. Data were collected in class during 2017–2018; participation was scored as an optional part of their credit in the subjects “Individual Differences” and “Environmental Psychology”. “Individual Differences” were studied by bachelor students in psychology as a mandatory course; non-psychology students studied the course “Individual Differences” as minors, that is, bachelor students, non-specialists in psychology, but the same program as psychology students. “Environmental Psychology” was an open elective course, attended by all people who were interested in this subject, that is, researchers, bachelors, master students of different specialties, and external students with higher education, independent of their specialties and levels.

The People and Plants questionnaire (PaP) was used to obtain information concerning attitudes toward the plant world [11,12]. Authors developed the questionnaire on a sample of Russians of different ages, urban citizens, who have had different experiences of interaction with flora and competence in the care of plants and flowers. Since plants are considered as a resource for the positive functioning of urban citizens, it was important that the validation group also consisted of urban citizens. In rural Russia, which is not surprising, consumer and pragmatic attitudes to plants prevails, which is meant for people’s survival, and therefore, psychotherapeutic or developmental effects are not expected in this social group (however, this may be the subject of a future study).

The questionnaire’s statements were initially extracted from essays in which people described their relationships with plants in free form. Then, after a series of checks on the items, items that did not give a proper distribution or were not loaded into the factor structure of the questionnaire were discarded from the initial pool. The questionnaire is a theoretically sound and empirically constructed tool for assessing the attitudes of people to plants; a separate article is devoted to its psychometric verification [11,12]. 

Now, PaP consists of five scales and includes 32 items describing different aspects of people-plants interaction. The joy scale (11 items) assesses positive emotions that appear when people observe plants and flowers or interact with them. The esthetics scale (8 items) emphasizes the beauty of plants. The practice scale (4 items) shows how experienced in taking care of plants an individual is. The closeness to nature scale (6 items) measures general positive attitudes to the plant world. Finally, the ecology scale (3 items) describes the value of plants in the context of ecological situations. Examples of items: “When my plants die, I always get upset” (joy); “I like to photograph plants and flowers” (esthetics); “I come back from every trip with a plant (seeds, seedlings, or cuttings)” (practice); “I feel a surge of positive, warm feelings while in the woods” (closeness to nature); “A person should take care of plants because life on Earth is impossible without them” (ecology). A 4-point Likert scale was used.

To investigate the dependent variable (the moral motives), we have chosen the moral motives theory by Janoff-Bulman that has become very popular among Russian scientists and, besides, is equipped with a convenient tool for measuring moral motives [13,14,15]. Janoff-Bulman considered morality as a system of rules that coordinate living in communities; as such, it involves behavioral regulation to optimize people’s existence as social beings. Her taxonomy of morality is based on the most fundamental psychological distinction in the motivation—approach versus avoidance, or behavioral activation versus inhibition. In recent years, this approach-avoidance distinction has proved his heuristic in understanding very different phenomena across psychology. 

The behavioral inhibition system, based on avoidance, is sensitive to punishment and negative consequences. The behavioral activation system, based on approach, on the contrary, is susceptible to positive results and rewards. Janoff-Bulman applied these differences to the moral sphere of living and distinguished between proscriptive and prescriptive morality. Hence, groups of moral motives identified by Janoff-Bulman differ concerning their contents and targets. Proscriptive morality (based on avoidance) focuses on what we should not do; it involves restricting a motivation to do something terrible and thus overcoming temptation or desire. In other words, proscriptive morality prevents harming, and the right behavior involves inhibition. Prescriptive morality (based on approach) focuses on what we should do; in contrast to inhibition, it requires establishing a motivation to do something good. So, prescriptive morality provides for well-being, and the right behavior involves activation and engaging in helpful practices. Janoff-Bulman emphasized that, overall, proscriptive moral regulation is condemnatory and strict, whereas prescriptive morality is commendatory and less strict. This may be explained by the negativity bias in psychology, which entails higher motivational potency of negative (vs. positive) outcomes, and is evident in the moral domain as well. Proscriptive regulation is sterner and more demanding than the prescriptive one.

Additionally, Janoff-Bulman identified three kinds of social target morality: distinct focuses of moral concern, from Self (personal), to other(s) (interpersonal), and the group (collective). The self-focus involves the impact of an individuals’ behavior on themselves. The interpersonal focus refers to moral concerns directed to another individual or individuals, and the group category means a focus on the collective (one’s group) as a whole [13,14].

In total, there are six moral motives. Since the theory is not yet complete, the search for precise terms for motives continues. Proscriptive motives are as follows: self-restraint (Self), not harming (other), social order (group). Self-restraint (moderation, self-protect) is focused on protecting the Self through behavioral inhibition and resisting temptations. Not harming (other-protect) involves proscriptions to not physically harm and to not harm by taking advantage of another through lying, cheating, stealing, and the like. Social order (communal solidarity, group-protect) contributes to conformity behaviors and group loyalty to maximize group cohesion.

Prescriptive motives are self-reliance (industriousness) (Self), helping/fairness (other), and social justice (group). Self-reliance (industriousness, self-provide) moral motive relieves the individuals’ burden on the group and increases the group’s resources. Helping/fairness (other-provide) emphasizes the role of personal responsibility for fair treatment at the interpersonal level. Finally, social justice (communal responsibility, group-provide) strengthens group bonds through a shared sense of responsibility.

The Moral Motives Model scale was used to measure moral motives. It consists of six scales and 30 items [13,14]. Examples of items: “Life is full of unhealthy attractions, so I must develop a strong sense of self-discipline and control” (self-restraint); “We should never steal from other people” (not harming); “It is harmful to society when people choose radically new lifestyles and ways of living (social order)”; “When things get tough, I apply myself and work even harder to overcome difficulties” (industriousness); “Treating others fairly is a clear sign of a good person” (helping/fairness); and “Increased economic equality is ultimately beneficial to everyone in the society” (social justice).

The Russian version of the Moral Motives Model scale was first used in 2018 and showed good psychometric properties; the full description of the Russian version of the tool is planned to be a separate publication [15].

Statistical analysis was performed using Statistica 8 (StatSoft Inc., Tulsa, USA).

## 3. Results

First of all, we have counted descriptive statistics regarding all variables investigated; after that, a correlation table was formed, and finally, we performed a multifactorial regression analysis. As not all of the variables met the normality criteria (but were near normality), we used non-parametric statistics. First of all, this applies to the practice scale that has a floor effect (no surprise that most urban students have not had much experience in interaction with the plant world). At the same time, our sample has a size sufficient for multifactorial analysis.

All scales used in the study demonstrated good reliabilities (see Table 1). Self-restraint and social justice motives have shown the highest variability among all the motives, whereas self-reliance—the lowest one. It seems to be caused by the specificity of our sample, that is, students from one of the most prestigious Moscow universities, where there is a very competitive and demanding environment. Thus, students are forced to rely on themselves and to be very persistent in achieving their goals.

As our data have shown, there are no gender differences in variables except social order motive, which score was higher in females (Mann–Whitney U = 4548.0, *p* = 0.02). It means that female students tend to protect their social groups, express communal solidarity, and demonstrate conformist behaviors more likely as compared to male students who are less dependent on the group and freer from social loyalty. As for the scores of attitudes toward the plant world, no differences were found. Based on these data, we performed further analysis without splitting the sample by gender. 

As expected, we have received many positive correlations between parts of attachment to the plant world and moral motives (see Table 2). Only the practice scale describing actual individuals’ experience in taking care of plants formed fewer connections with moral motives; nevertheless, it correlated positively with social justice, social order, and self-restraint. Maybe it was so because the sample consisted of urban citizens, mostly who might have never had any relations to plants or flowers. In line with this suggestion, the esthetics scale formed the highest number of strong links to all moral motives. It is easy to interpret, taking into account the content of this scale, reflecting individuals’ sensitivity to the beauty of plants, not only alive but painted or embroidered as well. Hence, the facts obtained give empiric support for Dostoevsky’s idea that beauty will save the world. Indeed, people who admire plants are ready to help their neighbors and not to harm them, keep social order, maintain social justice, hard work, and restrain their needs at the same time [16]. 

Joy and closeness to nature scales also correlated positively with all the moral motives, although the scores were not as high as in the case of other PaP variables. Nevertheless, these results show simultaneity of connectedness to nature, positive emotions, and moral experiences. Finally, the strongest connections were found between the ecology scale and moral motives (except social order). This means that the ecological world view, based on recognition of all living species, their interconnectedness, and interdependence, most strongly embodies the moral qualities of a person. Therefore, it may be worthwhile to pay attention to the systemic, holistic understanding of the living world in the process of teaching natural sciences in the first place. 

The next step of the analysis was the development of the multi-regression models showing a predictive role of various aspects of attachment to plants for moral motives (Table 3). Since joy, esthetics, and closeness to nature, as independent variables, correlated with each other highly, to avoid multicollinearity, three models for each dependent variable were constructed. The first model included esthetics, practice, ecology, and gender as the independent variables; the second model—joy, practice, ecology, and gender; the third model—practice, closeness to nature, ecology, and gender. Although most models were not very strong, they demonstrate that attachment to plants may contribute to moral motives. 

As was shown, all the moral motives except self-reliance are predicted by at least one of the aspects of attachment to plants (Table 3). Helping/fairness, social justice, and self-restraint were predicted by ecology which means respect for the whole system where people live. It is not surprising that this particular feature, being beyond egoistic values, contributes to three moral motives: people who are aware of the interdependence of living forms on Earth (ecology) are more likely to restrain their own needs, to not harm other people, to actively help others, and to maintain social justice. This fact is in line with ethical and philosophical theories we have based our research on; indeed, people who like plants as part of nature, tend to go beyond their personalities, focus on the interests of the living in general, which, in turn, means the absence of an egoistic world view. 

Helping/fairness, not harming, and social order are predicted by esthetics, which reflects individuals’ sensitivity to beauty and may be connected to more complex and nuanced higher motives. Love for nature strengthens the sense of harmony, the sense of beauty in people, which also elevates and ennobles the person, making him/her more moral at the same time. Social order is predicted by practice as well; people who have any experience in dealing with plants are more likely to take into account social rules compared with those who do not have such experiences and impressions. Furthermore, closeness to nature and joy, contrary to our expectations, did not predict any moral motives at all. Finally, four out of six motives, namely helping/fairness, not harming, social order, and self-reliance are predicted by gender as well, with higher scores in the female group. This result demonstrates, once again, that the careful attitude to nature and the world, as well as some conservatism, are more pronounced in females.

## 4. Discussion

The study was carried out by correlation design, with all the limitations of this method. Strictly speaking, distinguishing between dependent and independent variables was relative and answered the research question: Can attachment to the plant world contribute to the moral attitude towards people? Can flora be a resource for human moral elevation?

Results obtained show that they are indeed positively related; almost all moral motives are connected with all components of a positive attitude toward flora. This connection indicates that these phenomena overlap, especially strongly in the area of proscriptive morality. Perhaps there might be some common factors behind them. The fact that the so-called proscriptive motives are more strongly predicted by positive attitudes toward flora, as compared with prescriptive motives, is in full compliance with an observation by Janoff-Bulman regarding higher strength of proscriptive morality in general [13]. This means that proscriptive morality might have more shades of manifestation and connections with other variables. We would like to point out that it was the self-restraint motive that formed the links to all aspects of a positive attitude toward flora.

At the same time, prescriptive motives are weaker connected with the attitude to plants; other resources except flora may be used to stimulate them. Only the self-reliance motive, which is important for the actual achievement of goals, was not predicted by the attitude to plants at all. In the absence of similar results, we can only speculate that, perhaps, attachment to the plant world, especially among urban citizens, contributes to a non-pragmatic worldview, free from ambition and focusing on successes and achievements, which results in strengthening exact proscriptive morality. Of course, this assumption needs further research. 

Another noteworthy result is that the practice, as part of the attitudes to the world of flora, has formed the fewest connections. This is not surprising, as most respondents were citizens. Besides, to the extent that sample size allowed, it was discovered that the connection between attitudes toward flora is gender-sensitive, and the links obtained are stronger in females. This fact is easily interpreted, taking into account that urban women are more likely to be amateur plant breeders.

In some part, the results obtained are in line with previous data regarding the connection between environmental identity and empathy; in actuality, the feeling of connectedness to and rootedness in nature also predicts compassion toward people [10]. 

The results do not oppose ethical ideas of the reverence for life theory by Schweitzer [1] and deep ecology by Næss [2], who emphasized people’s opportunity in their self-actualization, owing to nature, to transcend beyond their survival and pragmatic goals, and arrive at the position of universalism. This position defines more tolerant attitudes to other people and even other forms of life [10]. Moreover, people may extend their willingness to protect and support other people, starting from attachment to plants [3,4]. At the same time, it has been shown that the strongest predictor of moral motives among all components of a positive attitude toward nature is ecology. In contrast, closeness to nature and joy did not predict moral motives at all. This fact makes us think that the natural and social identity of people may not be harmonized, and in this case, the respect for nature does not mean respect for the social reality and interests of the community. This makes it possible to identify a risk group among people who demonstrate exaggerated care for nature without understanding its connection with society. Also, we have received no connections between the joy of plants and moral motives, which means, again, that people who enjoy and admire plants are not necessarily moral. On the other hand, these outcomes show that love for plants is not synonymous with love for people, and the ennobling function of interacting with plants should not be overestimated. It is necessary to make some personal transcendence, so that to relate positively to both nature and people. 

The findings seem to be helpful in programs and training of moral development, as well as in ecological education where they may have applied value. Plants are a part of nature that is easiest to bring into the house or the office. That is why it is important to track all effects that plants have, not only on people’s health and psychological well-being, but also on their moral lives. Based on the results obtained, we can expect that the green offices cannot only increase the employees’ psychological well-being but also change the emotional atmosphere to warmer, if people are ready to restrain themselves and take into account the needs of others. 

## 5. Conclusions

As expected, positive attitudes to the plant world are positively related to moral motives of the broad spectrum, especially to proscriptive motives, and this connection is more prominent in the female group. Prescriptive motives, dealing with active goal achievements, are less sensitive to interaction with the plant world. To sum it up, communication with flora is accompanied by people’s morality, indeed. At the same time, it has become clear that love for nature in itself does not ennoble people, but only in combination with an ecological world view, which takes into account the interests of all living things, including people. 

Our study is not free of some limitations, however. 

The correlation design causes the first of them. Hence, we can confirm that the positive attitudes to the plant world are connected with human morality and may contribute to the moral motives, but we cannot follow this link as a causal one. To achieve more precise and evidential outcomes, we have to launch a longitudinal or experimental study.

The second limitation relates to the sample; we investigated only students and mostly females. However, to justify this sample, it should be noted that it is young urban intellectuals, not rural residents, who at the next stage of their lives become consumers of all kinds of psychotherapeutic interventions, including gardening, horticultural therapy, and other interactions with plants.

As for gender-sensitive results, they should be investigated again, in detail, in the more gender-balanced sample. In future research, it seems to be very promising to add the variable of experience in dealing with the plant world, and to invite more diverse groups regarding age, gender, and social strata. Moreover, it would also be useful to investigate other variables that describe the relationship to nature, such as the attachment to animals or environmental identity in general. Another area of research is the change of dependent and independent variables. Moral motives may be a more stable phenomenon than the attitude to plants, and therefore, can be decisive in attitudes to nature.

## Figures and Tables

**Table 1:** Descriptive statistics and reliabilities of all variables investigated.

Title 1	Mean	Minimum	Maximum	SD	Cronbach’s Alpha
Helping/fairness	5.14	1.00	7.00	0.97	0.69
Not harming	4.91	1.60	7.00	1.04	0.71
Social justice	4.18	1.00	7.00	1.08	0.72
Social order	4.17	1.00	7.00	0.99	0.79
Self-reliance	5.12	1.60	7.00	0.83	0.81
Self-restraint	4.57	1.00	7.00	1.19	0.74
Joy	2.27	0.00	4.00	0.85	0.75
Esthetics	2.63	0.00	4.00	0.78	0.76
Practice	1.56	0.00	4.00	0.68	0.71
Closeness to nature	2.85	0.00	4.00	0.84	0.85
Ecology	3.06	0.00	4.00	0.86	0.89

N = 257. Proscriptive motives are in grey.

**Table 2:** The connection between attitudes toward the plant world and moral motives (Spearman r).

Pap Scales	Helping/Fairness	Not Harming	Social Justice	Social Order	Self-Reliance	Self-Restraint
Joy	0.18; 0.004	0.19; 0.003	0.21; 0.001	0.15; 0.014	0.15; 0.019	0.20; 0.001
Esthetics	0.29; 0.000	0.26; 0.000	0.24; 0.000	0.26; 0.000	0.23; 0.000	0.25; 0.000
Practice	0.05; 0.382	0.12; 0.054	0.16; 0.009	0.19; 0.002	0.12; 0.059	0.19; 0.003
Closeness to nature	0.21; 0.001	0.17; 0.005	0.14; 0.023	0.14; 0.025	0.17; 0.005	0.19; 0.002
Ecology	0.27; 0.000	0.21; 0.001	0.23; 0.000	0.08; 0.231	0.16; 0.009	0.35; 0.000

N = 257. First number is a correlation index; second number is a level of significance.

**Table 3:** Predictive validity of the attitudes toward the plant world for moral motives.

Model	Predictor	R^2^	Βετα/ p	F	P of the Model
**Helping/fairness**					
1	Ecology	0.13	0.22/0.002	9.32	*p* < 0.000
	Esthetics		0.20/0.011		
2	Ecology	0.11	0.27/0.000	8.02	*p* < 0.000
	Gender		0.13/0.036		
3	Ecology	0.12	0.23/0.002	8.45	*p* < 0.000
**Not harming**					
1	Esthetics	0.10	0.22/0.006	6.67	*p* < 0.000
	Gender		0.15/0.014		
2	Ecology	0.08	0.14/0.047	5.60	*p* < 0.000
	Gender		0.17/0.006		
3	Gender	0.07	0.16/0.009	5.00	*p* < 0.000
**Social justice**					
1	Not significant				
2	Not significant				
3	Ecology	0.06	0.19/0.015	4.16	*p* < 0.003
**Social order**					
1	Esthetics	0.11	0.22/0.007	7.38	*p* < 0.000
	Gender		0.16/0.008		
2	Practice	0.08	0.20/0.016	5.35	*p* < 0.000
	Gender		0.17/0.003		
3	Practice	0.08	0.18/0.009	5.46	*p* < 0.000
	Gender		0.18/0.004		
**Self-reliance**					
1	Not significant				
2	Gender	0.05	0.12/0.046	3.14	*p* < 0.015
3	Not significant				
**Self-restraint**					
1	Ecology	0.13	0.30/0.000	9.56	*p* < 0.000
2	Ecology	0.13	0.32/0.000	9.53	*p* < 0.000
3	Ecology	0.13	0.36/0.000	9.76	*p* < 0.000

N = 256. Only significant connections are shown. Proscriptive motives are in grey.
